# Acute oral toxicity and anti-inflammatory activity of hydroalcoholic extract from *Lampaya medicinalis* Phil in rats

**DOI:** 10.1186/0717-6287-47-6

**Published:** 2014-03-26

**Authors:** Glauco Morales, Adrián Paredes, Alberto Olivares, Jaime Bravo

**Affiliations:** Departamento de Química. Facultad de Ciencias Básicas, Universidad de Antofagasta, Antofagasta, Chile; Laboratorio de Química Biológica, Instituto Antofagasta (IA), Universidad de Antofagasta, Antofagasta, Chile; Departamento de Acuicultura, Facultad de Recursos del Mar, Universidad de Antofagasta, Antofagasta, Chile; Departamento Biomédico, Facultad de Ciencias de la Salud, Universidad de Antofagasta, Antofagasta, Chile

**Keywords:** *Lampaya medicinalis*, Hydroalcoholic extract, Acute oral toxicity, Histology, Hematology, Anti-inflammatory, Carrageenan

## Abstract

**Background:**

Algesia and inflammation are related with several pathological conditions. It is known that many drugs available for the treatment of these problems cause unwanted side effects. This study was aimed at evaluating acute toxicity and anti-inflammatory activity of *Lampaya medicinalis* Phil. (Verbenaceae) widely used in the folk medicine of Northern Chile against rheumatism, arthritis and body joints pain.

**Results:**

Oral administration of hydroalcoholic extract (HAE) at the highest dose of 3000 mg/ Kg body weight resulted in no mortalities or evidence of significant behavioral changes. Histological examination revealed normal architecture and no significant adverse effects were observed on the liver, kidney, heart, lung or ovaries and testicles. The results suggest that the oral administration of hydroalcoholic extract (HAE) from *Lampaya medicinalis* did not produce any toxic effect in rats. Hydroalcoholic extract (HAE) significantly inhibited the carrageenan-induced rat paw edema in dose – response relationship, at test doses of 37.5, 75, 150 and 300 mg/Kg body weight. Maximum inhibition (61.98 ± 2.69%) was noted at 300 mg/Kg after 2 h of drug treatment carrageenan induced paw edema, whereas indomethacin produced 47.90 ± 1.16% of inhibition. The inhibitory values of edema at 3 h postcarrageenan were 31.04±0.75%, 40.51 ± 2.36%, 48.97 ± 1.14% and 56.87 ± 0.41% for 37.5, 75, 150, and 300 mg/kg of extract respectively. Indomethacin (10 mg/Kg) gave a percentage inhibition of 49.44 ± 1.44. HAE (300 and 150 mg/kg) induced an anti-inflammatory effect greater than (or comparable) with the effect of indomethacin from 2nd to 4th hours of the experiment.

**Conclusions:**

Our results reveal for first time that compounds contained in the hydroalcoholic extract of *Lampaya medicinalis* Phil exert anti-inflammatory effect and the oral administration is safe and non toxic up to dose level 3000 mg/kg body weight. The anti-inflammatory activity may be associated with the presence of flavonoids. These findings also justify the traditional use of the plant for treating pain.

## Background

Medicinal plants are popular remedies used by a majority of the world´s population. The efficacy of medicinal plant in the management of diseases is indubitable. The World Health Organization estimated that 80% of the population of developing countries, continues to use traditional medicine in primary medical problems. Several plants are typically used without considering the toxicity and pharmacological aspects. The toxicity herbal preparation is usually unknown and the population does not care, believing that if the material has being used so far, it should be devoid of toxicity [1].

Information on the toxicity of the plants is very important as baseline before further exploring its development as a new herbal medicine [2].

Standard experimental methods are useful for the validation of ethnopharmacological knowledge regarding herbal medicine. Many studies are carried out to validate the use of medicinal plants in an effective and safe way [3, 4].

Acute toxicity studies are commonly used to determine signs of toxicity and effects on biochemical, hematological and histological parameters.

Chronic inflammation diseases remain one of the world´s major health problems. Inflammation has become the focus of global scientific research because of its implication in virtually all human diseases [5]. Since conventional anti-inflammatory drugs have not been successful in some inflammatory processes, there is an urgent need to have new and safe anti-inflammatory agents [6]. Attention is being focused on the investigation of the efficacy of plants-based drugs used in the traditional medicine [6]. Inhibition of carrageenan-induced inflammation has been shown to be highly predictive of anti-inflammatory drug activity.

Andean High Plateau in Northern Chile, known as “Puna atacameña”, is a particular biotope characterized by varying altitudes of between 3000–4200 meters above sea level, a very low relative humidity, no rains, cloudless skies during most of the year. Around 5000 peoples live in this peculiar ecological system and they use medicinal plants as curatives or palliatives of health problems because the plants are recognized as a traditional way to treat ailments and diseases.

*Lampaya medicinalis* Phil (Verbenaceae) commonly known as “lampaya”, is a small bush, with a height of 80 – 100 cm that grows in the “Puna atacameña” in Northern Chile.

There is little or no information about the chemical composition or medicinal properties of this plant in the scientific literature; however, oral reports from herbal medical practitioners indicate that the infusion of the plant is usually prepared and given for treatment of colds, stomach pain, urinary bladder discomforts, as antitussive, and against rheumatism, arthritis and body joints pain [7]–[10]. It has been reported that agents isolated from *Lampaya hieronymi* exhibit anti-inflammatory activity [11].

The objective of this research was to validate the use of *Lampaya medicinalis* for folklore medicine, therefore anti-inflammatory potential of the hydroalcoholic extract from on carrageenan-induced rat paw edema was explored, toxicity was evaluated using an acute oral toxicity test in rats under the Organization for Economy Cooperation Development guidelines [12, 13] and the phytochemical screening of hydroalcoholic extract was evaluated [14].

## Results

### Acute toxicity study

#### Lethality and behavioral analysis

In this 14-days period of acute toxicity evaluation, rats given HAE from *Lampaya medicinalis* leaves in a single dose level of 3000 mg/kg body weight, showed no mortality and none of them showed any symptom of toxicity. The behavioral pattern of animals was observed first 5 h, 12 h and every day for 14 days after the administration, and the animals in both vehicle treated and extract treated groups were normal and did not display significant changes in general behavior. This visual observation showed no significant changes in behavior, skin effect, breathing, defecation, postural abnormalities, impairment in food intake and water consumption and yellowing or loss of hair, compared to negative control group (rats no treated). Neither mortality, nor tremors nor convulsions were noted after 14 days of treatment.

#### Organ and body analysis

The mean of rats body weight was measured on a daily basis for 14 consecutive days. No statistically significant differences were shown among group of rats treated compared with negative control group. The last day of treatment, animals were anesthetized and blood collected by cardiac puncture. The rats were sacrificed and liver, heart, kidney, lung and sexual organs were collected. There were no significant changes in relative organ weight between both control and treated groups. The relative liver weights were 2.70 ± 0.03 g/100 g of b.w. and 2.76 ± 0.02 g/100 g of b.w, for control and treated group, respectively. Values for kidneys were 0.37 ± 0.02 g/100 g of b.w. and 0.39 ± 0.02 g/100 g of b.w, for control and treated group, respectively. The results revealed that the essential organs as liver and kidney were not adversely affected throughout the treatment. Macroscopic analysis of target organs of treated animals did not show significant changes in color, volume and texture when compared with the control group.

#### Biochemistry analysis

After administering a dose of 3000 mg/kg b.w. of HAE from *L. medicinalis,* serum levels of alanine aminotransferase (ALT) were 57.40 ± 2.79 (IU/L) and 11.00 ± 1.41 (IU/L) for the control group and the treated group, respectively. While the levels for aspartate aminotransferase (AST) were 208.60 ± 4.35 (IU/L) and 170.49 ± 3.92 (IU/L) for the control group and the treated group, respectively. In both cases the differences are not statistically significant.

#### Histopathology of liver, heart, kidney, sexual organs and lung

**Figure 1:**
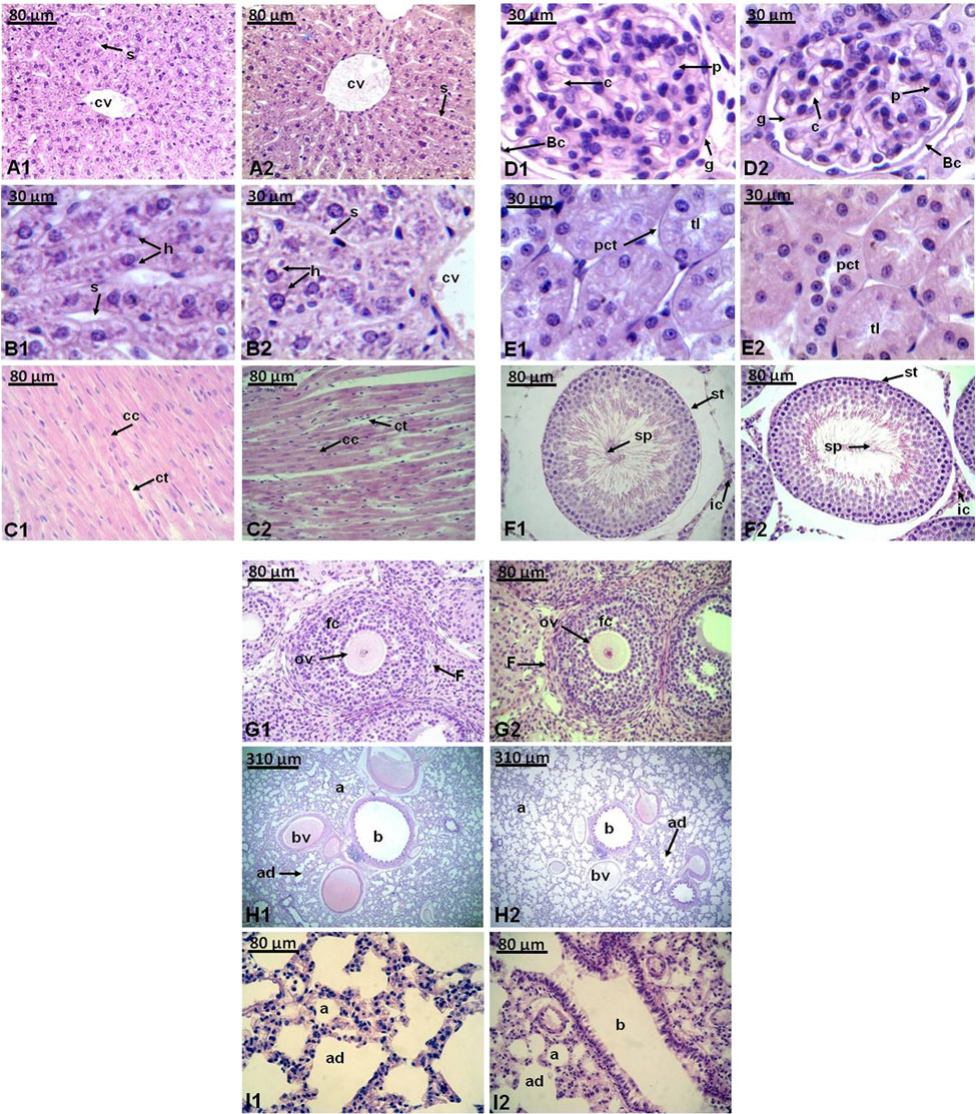
**Representative photomicrographs of histological preparation (0.5 µm), stained with hematoxylin and eosin of liver** **(A1,A2,B1,B2)** **, heart** **(C1,C2)** **, kidney** **(D1,D2,E1,E2)** **, testis** **(F1,F2)** **, ovaries** **(G1,G2)** **and lung** **(H1,H2,I1,I2)** **of rats controls (1) and treated (2) with HAE of** ***Lampaya medicinalis*** . Abbreviation: cv (central vein), s (sinusoids), h (hepatocyte), cc (cardiac muscle cell), ct (connective tissue), g (glomerulus), p (podocyte), Bc (Bowman´s capsule), c (capillarie), pct (proximal convoluted tubule), tl (tubular lumen), sp (spermatozoids), st (seminiferous tubule), ic (interstitial Leydig cell), ov (oocyte), F (follicle), fc (follicular cell), b (bronchioli), a (alveoli), ad (alveolar duct), bv (blood vessel). The number above the bar indicates the amplification.

The microscopic structures of the organs described in Figure 1 shows unnoticeable differences between the control and test group. The microscopic examination revealed that liver, heart, kidney, sexual organs and lung from the extract treated rats did not show any alteration in cells structure or any unfavorable effects when viewed under the light microscope using multiple magnification power. The structure or coordination of cells of organs treated with extract of *Lampaya medicinalis* were similar compared with the control group. The cellular structures compared in each tissue were: central vein, sinusoids and hepatocytes in liver, cardiac muscle cell and connective tissue in heart, glomerulus, podocyte, Bowman´s capsule, capillarie, proximal convoluted tubule and tubular lumen in kidney, spermatozoids, seminiferous tubule and interstitial Leydig cell in testis, oocyte, follicle, and follicular cells in ovarium tissue and bronchioli, alveoli, alveolar duct and blood vessel in lung.

### Rat paw edema assay

The hydroalcoholic extract (HAE) of *Lampaya medicinalis* was very effective in causing inhibition of the paw volume in the carrageenan-induced paw edema in rat. The anti-inflammatory activity at test doses of 37.5, 75, 150 and 300 mg/kg body weight of HAE is presented in Table 1 with the average volume of carrageenan-induced rat paw edema. The percent protection of inflammation is presented in Table 2. The injection of carrageenan in paw created an inflammatory edema which increased gradually. HAE significantly inhibited the carrageenan-induced rat paw edema in dose - response relationship. The inhibitory values of edema at 3 h postcarrageenan were 31.04 ± 0.75%, 40.51 ± 2.36%, 48.97 ± 1.14% and 56.87 ± 0.41% for 37.5, 75, 150, and 300 mg/kg of extract respectively. Indomethacin (10 mg/kg) gave a percentage inhibition of 49.44 ± 1.44%. HAE showed a significant effect even at the smallest dose (37.5 mg/kg), which inhibited paw edema by 30.16 ± 0.91% at the 2^nd^ hour after carrageenan administration.

**Table 1:** Volume of paw edema

Time (hours)	Volume of paw edema (mL)					
	Control	HAE				Indomethacin (10 mg/kg)
		300 mg/kg	150 mg/kg	75 mg/kg	37.5 mg/kg	
1	1.86 ± 0.32	0.96 ± 0.22	1.39 ± 0.35	1.53 ± 0.29	1.58 ± 0.16	0.91 ± 0.02
2	2.63 ± 0.10	1.00 ± 0.27	1.43 ± 0.17	1.39 ± 0.18	1.84 ± 0.58	1.37 ± 0.11
3	2.69 ± 0.23	1.16 ± 0.09	1.37 ± 0.22	1.60 ± 0.15	1.86 ± 0.36	1.36 ± 0.44
4	1.82 ± 0.21	0.81 ± 0.26	1.12 ± 0.45	1.46 ± 0.71	1.53 ± 0.78	1,19 ± 0.20
5	1.21 ± 0.17	0.89 ± 0.18	0.94 ± 0.19	1.04 ± 0.97	1.07 ± 0.82	0.77 ± 0.17

Results are shown as mean edema volume ± SEM (n=6) compared with control group. Statistical significance according to Student´s *t* test. P < 0.05.

**Table 2:** Percentage of inhibition inflammation

Time (hours)	% inhibition of inflammation				
	HAE				Indomethacin (10 mg/kg)
	(300 mg/kg)	(150 mg/kg)	(75 mg/kg)	(37.5 mg/kg)	
1	48.38 ± 0.13	25.05 ± 1.13	17.63 ± 1.12	15.04 ± 0.53	51.07 ± 0.30
2	61.98 ± 2.69	45.49 ± 0.75	47.13 ± 1.06^*^	30.16 ± 0.91	47.90 ± 1.16
3	56.87 ± 0.41	48.97 ± 1.14^*^	40.51 ± 2.36	31.04 ± 0.75	49.44 ± 1.44
4	55.49 ± 1.22	38.46 ± 1.20	19.78 ± 0.79	15.93 ± 0.22	34.61 ± 0.81
5	26.45 ± 1.07	22.31 ± 0.75	14.05 ± 1.02	11.57 ± 0.61	36.36 ± 0.42

Results inhibition percentage ± SEM (n=6) when compared with control group (Indomethacin). Statistical significance according to Student´s *t* test (P < 0.05).

^*^Represent values not significant when compared with control.

HAE at dose of 300 mg/kg exhibite an anti-inflammatory activity that became significant 1 h after the injection of carrageenan and was maintained all along the experiment. Maximum inhibition, 61.98 ± 2.69%, was noted at 300 mg/kg after 2 h of drug treatment carrageenan induced paw edema, whereas indomethacin produced 47.90 ± 1.16% of inhibition. HAE (300 and 150 mg/kg) induced an anti-inflammatory effect greater than (or comparable) with the effect of indomethacin from 2^nd^ to 4^th^ hours of the experiment.

### Phytochemical analysis

Phytochemistry screening of the extracts revealed the presence of flavonoids, tannins, phenols and carbohydrates and reducing sugar.

## Discussion

With the resurgence of the use of medicinal plants, scientific studies have become imperative to validate the folkloric use. In acute toxicity studies, a single dose of drug is given in large quantity to determine immediate toxic effect. These studies are commonly used to evaluate LD_50_, signs of changes in behavior, effects on biochemical parameter and histopathology assessment of the essential organs as liver and kidney [15].

*Lampaya medicinalis* is a plant used in altoandean traditional medicine at northern Chile for the treatment of various ailments. The acute oral toxicity of hydroalcoholic extract from leaves of *Lampaya medicinalis* was determined in the present study. 12 healthy rats from both sexes were employed to observe the toxicity effects of HAE. In this study the results showed that in a single dose there are no adverse effects of HAE of *Lampaya medicinalis*, indicating that the medium lethal dose (LD_50_) is higher than 3000 mg/kg for rats. All animals treated with HAE survived beyond the 14 days observation period. The results revealed that the weights of liver and kidney were not adversely affected throughout the treatment. Macroscopic analysis of target organs of treated animals did not show significant changes in color, volume and texture when compared with the control group. Transaminases AST and ALT are well known enzymes used as biomarkers predicting possible toxicity. Increase in the level of AST and ALT in blood reflects the structural and functional dysfunction of hepatocellular membrane or cell rupture, and thereby indicate liver damage associated with tissue injury and reflection of hepatic toxicity. In the present study, AST and ALT levels were not affected by HAE, when is compared treated and controls animals. The lack of significant alterations in the levels of transaminases ALT and AST, good indicators of liver functions, suggests that acute ingestions of *L. medicinals* extract does not alter the hepatocytes of the rats, and, furthermore, the normal metabolism of the animals.

Histological examination is the golden standard for evaluating treatment related pathological changes in tissues and organs. Histological analysis of liver, heart, kidney, sexual organs and lung (Figure 1) showed an indistinguishable cellular architecture of the animals treated and the control group no treated, indicating that the HAE of *Lampaya medicinalis* did not adversely affect the morphology of organs of rats. The liver is the main target organ of acute toxicity where exposed to the foreign compounds which may or not to be hepatotoxic to the rats. In this study, the histological examination revealed that there was no potential toxicity or cellular lesions. Furthermore no necrosis or inflammation reaction was observed and the cell arrangement was similar to the organs of the control rats and treated.

Therefore, it can be suggested that HAE did not interact with the target cells or change the biological systems of the animals.

The most commonly used test to study new anti-inflammatory agents evaluates the ability of the chemical material to reduce local edema induced in the rat paw by injection of an irritant agent [16]. Irritant agents commonly used are: histamine, dextran, serotonin, carrageenan [17]. The development of edema in the paw of the rat after injection of carrageenan has been accepted as a useful tool for investigating systemic anti-inflammatory agent [18]. The inflammation model of a carrageenan induced edema is usually used to assess the activity of natural products in resisting the pathological changes associated with acute inflammation [19, 20]. Inflammation induced by carreegenan is acute, non immune, well-researched, and highly reproducible. Cardinal signs of inflammation – edema, hyperalgesia, and erythema – developed immediately after subcutaneous injections a result of the action of proinflammatory agents and reactive oxygen and nitrogen species [21].

The inflammatory response is usually quantified by increase in paw size (edema) which is maximal around 5 h postcarregenan injection. The results obtained from the carrageenan-induced paw edema shows that paw edema was markedly inhibited by the oral administration of the hydroalcoholic extract of *Lampaya medicinalis* (HAE) in dose - response relationship. The effect observed, which was time-dependent, lasted for at least 4 h with the two highest doses. The inhibitory values of edema at indicating that the extract is orally active at doses ranging from 37.5-300 mg/kg and can inhibit a acute inflammatory process [22].

## Conclusions

Hydroalcoholic extract of *Lampaya medicinalis* Phil leaf up to the dose level 3000 mg/kg body weight did not produce any toxic effects or deaths; the extract was well tolerated by the rats. It did not alter body weight, feed and water consumption. The organ weight, biochemical and hematological analysis did not show changes in any of the parameters examined in animals of both sexes. The acute oral administration of the hydroalcoholic extract of *Lampaya medicinalis* Phil leaf was safe and not toxic in a single dose.

Finally, the results of present study shows that *L. medicinalis* has anti-inflammatory activity. Therefore, the practice of drinking the infusion of the plant for treatment of rheumatism, arthritis and body joints pain by traditional medical practitioner is not totally out of place. There is a need for further studies in order to isolate the anti-inflammatory ingredients in the plant and determine their mechanism of action.

## Methods

### Plant material

Leaves and aerial parts of *Lampaya medicinalis* Phil were collected at Socaire in Northern Chile (23° 36´40 s S; 67° 50´33 s W, 3230 m above sea level). The material was identified by Professor Roberto Rodriguez, Facultad de Ciencias Biológicas y de Recursos Naturales de la Universidad de Concepción and voucher specimens are kept at the Herbarium of Universidad de Concepción, Chile.

### Preparation of the hydroalcoholic extract (HAE)

The air-dried leaves of *Lampaya medicinalis* Phil (1.2 kg) were chopped and exhaustively extracted with EtOH:H_2_O (1:1, 10 L) during one week at room temperature. The EtOH:H_2_O extract was then filtered, evaporated under reduced pressure and freeze – dried to obtain a viscous mass of dark green extract (HAE). The yield of the lyophilized aqueous - ethanol solution was 12.5% (w/w). The dried extract was sealed in a bottle and stored in the refrigerator at 4°C until further use. Just prior to the biological testing the extract was dissolved in saline to prepare a stock solution of 200 mg/mL.

### Target animals

Sprague–Dawley rats of both sex weighing 180–220 g were obtained from animal house of Facultad de Ciencias de la Salud de la Universidad de Antofagasta.

The animals were maintained on a standard pellet diet with free access to water and housed under controlled room temperature at 22 ± 2°C with lighting from 8:00 to 20:00.

The animals were used taking into account international principles and local regulation concerning the care and use of laboratory animals. All experimental protocols were approved by the Comité de Etica de la Universidad de Antofagasta.

### Acute toxicity study

The acute oral toxicity study was performed as per OECD-423 guidelines [12], 12 animals in 2 groups of 6 rats/group (3 males and 3 females) were used for the study. Drinking water and food were provided *ad libitum*, except for the short fasting period where the water was in free access but no food was provided 12 h before treatment. Stock solution of HAE, 200 mg/mL, was made in saline. Following the fasting period, body weight of each animals were determined and the dose was calculated in reference to the body weight as volume of the stock solution. HAE at dose of 3000 mg/kg body weight were administrated by gastric gavage using a suitable intubation cannula in the treatment group. Other 3 males and 3 females were allotted saline and were regarded as control group. After the substance has been administered, food may be withheld for a further 2–3 hours.

All the animals were observed after the administration of the HAE. Thereafter, observation were made at every hour for 5 h, then at 12 h and every day for 14 days. All observation were systematically recorded, with individual records being maintained for each animal. The observation of the cage includes the evaluation the skin and the eyes; the breathing effects; the effects of the autonomous system as salivation, diarrhea, micturition; the central nervous effects, as tremors and convulsion. At the end of the acute toxicity study, all animals were fasted 12 h and then, under a soft anesthesia were sacrificed and blood samples collected by heart puncture. The blood samples were centrifuged to 3000 rpm during 15 min. Serum samples were analyzed immediately for the determination of aspartate aminotransferase (AST) and alanine aminotransferase (ALT) by standard enzymatic methods using commercially available kit from Sigma-Aldrich.

Heart, kidneys, liver, lung, testes and ovaries of all animals were dried with filter paper, weighed using a analytic balance, and the relative weights expressed as g/100 g of body weights. The morphology was macroscopically observed for signs of toxicity. Samples of organs isolated from each individual were dehydrated by serial ethanol solution and enclosed with paraffin wax. Micrometer sections (5 μm) cut with microtome were stained with hematoxylin and eosin and were examined under a light microscope, photomicrographs of the samples were recorded.

### Rat paw edema assay

The carrageenan-induced hind paw edema test was perfomed according to [18, 23]. Sprague–Dawley rats of 180–220 g body weight divided into 6 groups with each group containing 6 rats. The control group and the reference group received normal saline (0.9% NaCl, 2 mL/kg) and indomethacin (10 mg/kg), respectively. While the test groups were treated with 37.5, 75, 150 and 300 mg/kg body weight of extract, respectively. Saline, extract and indomethacin were all administered orally. Carrageenan-induced inflammation was produced by injection of 0.1 mL of a freshly prepared 1% λ carrageenan suspended in sterile physiological saline into the right hind foot of each rat under the subplantar aponeurosis. The control, reference and the test groups were treated orally with saline, indomethacin and the extract 1 h before carrageenan injection. Paw volume was measured by a digital water plethysmometer (Ugo Basile, model 7140, Italy), before the treatment *(V*_*0*_*)* and 1,3,4,5 h after carrageenan injection *(V*_*t*_*)*. The increase in volume was taken as the volume of edema and was determined for each rat *(V*_*0*_*)*. The percent of edema inhibition in treated animals versus control no treated was calculated by the following equation [24, 25].

where D represents the edema volume after extract was administered to the rat and C represents the edema volume in control group.

### Phytochemical screening

Hydroalcoholic extract freeze-dried of *Lampaya medicinalis* was phytochemical screened in order to detected the presence (or absence) of alkaloids (Draggendorf and Meyer reagent), carbohydrates and reducing sugar (Molisch and Fehling reagent), flavonoids (Shinoda test), tannins and phenols (iron chloride) and terpenoids (Lieberman-Burchard test) [14].

### Statistical analysis

Results were expressed as the mean ± SEM. Treated groups were compared with the controls for statistical significant differences (p < 0.05) using paired Student´s *t*-test.
